# Modified Posterior Scleral Reinforcement as a Treatment for High Myopia in Children and Its Therapeutic Effect

**DOI:** 10.1155/2019/5185780

**Published:** 2019-01-22

**Authors:** Zequn Miao, Luojia Li, Xiaoli Meng, Lili Guo, Di Cao, Yanlei Jia, Dongmei He, Lvzhen Huang, Lejin Wang

**Affiliations:** ^1^Center of Optometry, Department of Ophthalmology, Peking University People's Hospital, Beijing, 100044, China; ^2^Beijing Key Laboratory of Diagnosis and Therapy of Retinal and Choroid Diseases, Beijing, 100044, China; ^3^Department of Ophthalmology, Zhongguancun Hospital of Beijing, Beijing, 100080, China; ^4^C-MER (Beijing) Dennis Lam Eye Hospital, Beijing, 100123, China; ^5^Shandong Zaozhuang Municipal Hospital, Zaozhuang, 277101, China; ^6^Department of Ophthalmology, Chaoju Hospital, Baotou, 014060, China

## Abstract

**Purpose:**

To investigate the safety and therapeutic effect of a modified posterior scleral reinforcement (PSR) in treating high myopia.

**Methods:**

A total of 85 highly myopic eyes in 47 children (6.3±3.6 years of age, range from 3 years to 15 years) who underwent this modified PSR were included in this study. Axial length, refractive error, best-corrected visual acuity (BCVA), macular scans, and adverse events were recorded before the operation (as a baseline) and in postoperative reviews taken yearly for 5 years.

**Results:**

This was a 5-year research: 50% of the children (20 children, 40 eyes) participated in the 6-month review, 41% of the children (17 children, 33 eyes) participated in the 1-year review, 26% of the children (11 children, 21 eyes) participated in the 2-year review, 16% of the children (7 children, 13 eyes) participated in the 3-year review, 13% of the children (5.3 children, 11 eyes) participated in the 4-year review, and 8% of the children (3.3 children, 7 eyes) participated in the 5-year review. Compared with the baseline, axial elongation was significantly changed (*P*<0.05) over the 5-year period in all of the children: 6-month (*P*=0.003), 1-year (*P*=0), 2-year (*P*=0), 3-year (*P*=0), 4-year (*P*=0), and 5-year (*P*=0). The axial length was extended. No significant difference was found in refractive error between measurements taken at baseline and at the 5-year postoperative visit in all of the children: 6-month (*P*=0.51), 1-year (*P*=0.50), 2-year (*P*=0.46), 3-year (*P*=0.56), 4-year (*P*=0.30), and 5-year (*P*=0.16). There were significant differences in BCVA between measurements taken at baseline and at the postoperative 5-year visit in all the children: 6-month (*P*=0), 1-year (*P*=0), 2-year (*P*=0), 3-year (*P*=0), 4-year (*P*=0), and 5-year (*P*=0). BCVA improved in 71 eyes (83.52%), remained stable in 14 eyes (16.47%), and declined in none of the children. No serious adverse events occurred before the operation and during the 5-year follow-up period.

**Conclusion:**

This modified PSR could be a therapeutic treatment for high myopia.

## 1. Introduction

Myopia has become a major public health concern, and there is evidence showing a shocking increase in its prevalence [1]. According to the literature, the prevalence of myopia has dramatically increased in developed countries in East and Southeast Asia [2, 3]. Morbidity statistics show that 80%-90% of high school children are myopic in urbanized East Asia and that 10%-20% of them eventually develop high myopia [2, 3]. Among the myopic children, high myopia is the most worrying factor [4]. The most obvious characteristic of high myopia is gradual growth in axial length, which increases rapidly in childhood and adolescence and then grows out of control in adulthood [5]. People suffering from high myopia may exhibit degenerative changes caused by the progressive elongation of the eyeball. These changes include sclera thinning, the formation of posterior staphyloma, fundus lesions (such as retinochoroidal atrophy), choroidal neovascularization (CNV), macular retinoschisis, a macular hole, and retinal detachment [6]. High myopia is a major cause of legal blindness worldwide and causes the most serious damage to vision in Asian countries [7–9]. Therefore, the increasing prevalence of high myopia in younger individuals indicates that there is an urgent need for effective treatments that can stop its progress as well as strategies aimed at preventing the occurrence of myopic complications [4].

A large number of treatments have been applied to halt myopia progression in children with low or medium myopia. While these include pharmacological intervention and contact lenses, these therapies achieve little progress in high myopia because axial growth progresses so quickly in these children [4]. Posterior scleral reinforcement (PSR) has been demonstrated to be an effective surgical method for slowing the elongation of the eyeball and preventing high myopic complications [10–16]. Currently, two main types of PSR are performed in the clinic: Snyder-Thompson PSR and Nurmamedov NN PSR [17–19]. The Snyder-Thompson PSR caused relatively severe surgical trauma and it is difficult for surgeons to obtain the surgical skills needed for it, but its advantage was also obvious since patients acquired good prognosis [20]. The Nurmamedov NN PSR, on the other hand, required fewer operating skills and caused less trauma, but the prognosis was poorer [10, 17]. Based on experience in previous PSR surgeries, Dr. Lejin Wang (People's Hospital) modified the PSR protocol that further simplifies the surgical procedures and minimizes operative injury.

## 2. Subjects and Methods

### 2.1. Subjects

This study is a retrospective analysis of 47 children (30 male, 17 female) (Table 1) with high myopia that was treated with a modified PSR protocol at our hospital between January 2011 and January 2018. The study was approved by the Medical Ethics Committee of Peking University and conducted in accordance with the Declaration of Helsinki tenets for research involving human subjects. Informed consent was obtained from all included children after a thorough discussion about both the desired positive outcomes and the potential adverse events of the PSR procedure.

The inclusion criteria applied in this study included high myopia, which was defined as a myopic refractive error ≥ 5.0 diopters (D) and an axial length ≥ 24 mm in subjects aged<8 years with a best-corrected visual acuity (BCVA) inferior to that observed in age-matched children. Children were excluded from the study if they had other ocular diseases that could affect visual function (e.g., nystagmus, glaucoma, lens abnormality, ocular trauma, a macular hole, macular atrophy, retinal detachment, and choroidal neovascularization), had systemic disorders that could interfere with the results, or had undergone another ocular surgery, such as refractive surgery, scleral buckle procedure, PSR, or vitrectomy.

### 2.2. Methods

#### 2.2.1. Surgical Procedures

**Figure 1:**
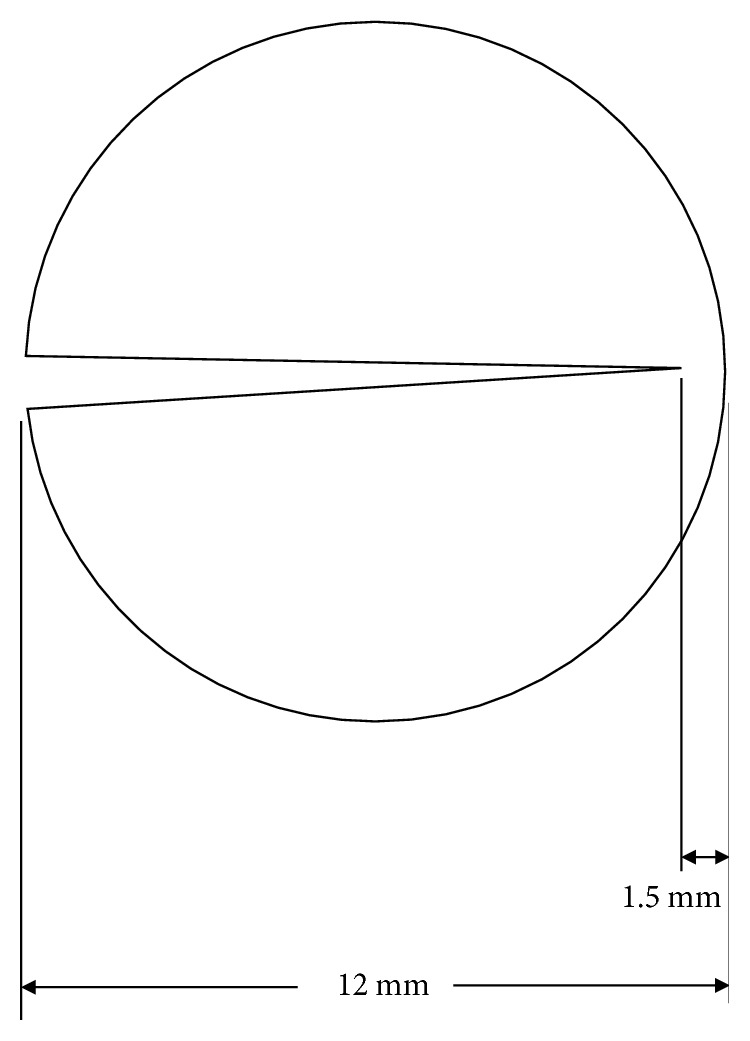
*Characterization of the scleral buckle.* Picture of a round bivalve scleral buckle, which was made by cutting a round scleral buckle out along the direction of the diameter while maintaining the root at 1.5 mm.

All operational procedures were conducted by the same surgeon (Dr. Wang). This modified PSR was performed under general anesthesia. At the very beginning of the surgery, a round bivalve scleral buckle (obtained from a donor sclera) with a diameter of 10-12 mm was made by cutting a round scleral buckle out along the direction of the diameter while maintaining the root at 1-1.5 mm (Figure 1). Before use, the ready-made bivalve buckle was reactivated by immersing it in 0.9% normal saline (NS) for 10 mins.

**Figure 2:**
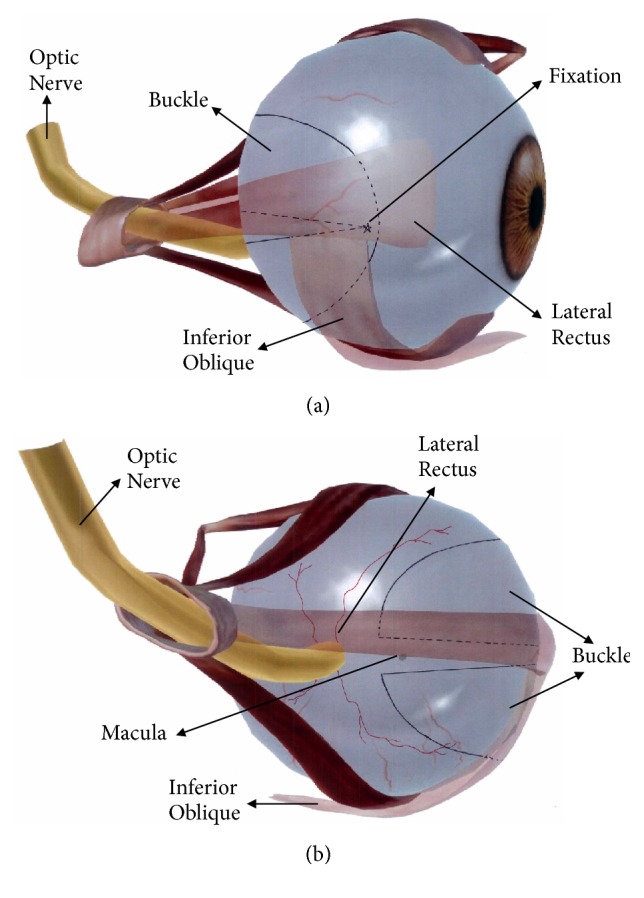
*Position of the scleral buckle.* (a) Temporal side of the eyeball. Half of the buckle was placed underneath the inferior oblique, while the other half was placed beneath the lateral rectus. (b) Back side of the eyeball. The representative picture showed relationship among the positions of the inferior oblique, lateral rectus, macula, and scleral buckle. The distance of the half-round scleral buckles was approximately 3 mm without compressing the optic nerve.

First, a temporal bulbar conjunctival incision was made approximately 2 mm from the lower corneal limbus. Second, the inferior oblique and lateral rectus muscles were identified, isolated, and exposed. The two muscles were maneuvered by strabismus hooks while the eyeball was pulled towards the superior nasal side so that the lower temporal part of the sclera could be exposed. Third, a traction suture was placed with 8-0 black silk at the root of the round bivalve scleral buckle. After fixing the buckle's root to the muscle insertion point at the anterior end of the inferior oblique, half of the buckle was placed underneath the inferior oblique (corresponding to the lower peripheral macular), while the other half was placed beneath the lateral rectus (corresponding to the upper peripheral macula). This procedure was accomplished by flattening the scleral buckle with the help of strabismus hooks. Finally, the location of the scleral buckle was checked. Its position relative to the buckle and optic nerve should, in particular, be tested with a strabismus hook. The distance was kept at approximately 3 mm to ensure that each part of the half-round scleral buckle covered the posterior staphyloma without compressing the optic nerve (Figure 2). The conjunctiva was closed with 8-0 absorbable sutures. After surgery, 0.5% levofloxacin and 0.1% fluorometholone eye drops were administered four times per day for two weeks.

### 2.3. Outcome Measures

All children underwent comprehensive ophthalmologic examinations, which included visual acuity, intraocular pressure, axial length, refractive error, best-corrected visual acuity, slit-lamp examination, and fundus examinations at baseline and at every postoperative follow-up visit (0.5, 1, 2, 3, 4, and 5 years after PSR) (Table 2).

#### 2.3.1. Axial Length

Axial length was measured with an IOL Master (Carl Zeiss Meditec, Dublin, CA, USA) (signal-to-noise ratio: >100). The statistical outcomes were measured five times, and the mean values were recorded.

#### 2.3.2. Refractive Error

Refractive error was measured with an autorefractor (ARK-700A, NIDEK, Japan) and a scientific subjective refractor (SSC-330, NIDEK Co., Ltd., Aichi, Japan). Subjective cycloplegic refraction was assessed by experienced optometrists approximately 30 min after 1 drop of tropicamide 0.5% was administered 3 times at 5 min intervals. These data are presented as the spherical equivalent (SE). The statistical outcomes were measured five times, and the mean values were recorded.

#### 2.3.3. Best-Corrected Visual Acuity

BCVA was measured with the Snellen acuity test “E,” and the results were converted to LogMAR for statistical analysis.

#### 2.3.4. Anatomical Outcomes

An extraocular muscle examination was performed by the surgery doctors and included eye movements and strabismus checks. The ocular anterior segment was assessed with a slit-lamp, and postoperative adverse events, such as conjunctival injection, edema, and inflammatory response, were recorded. The fundus was examined after the pupil was dilated, and macular scans were observed by indirect ophthalmoscopy (Zeiss). Intraocular pressure (IOP) was measured by a noncontact tonometer (Canon TXF).

### 2.4. Statistical Analysis

Postoperative axial length, refractive error, and BCVA were recorded and compared with preoperative measurements using a mixed model with repeated measures at each follow-up visit. The factors that might be associated with myopic progression, such as gender and age, were also taken into consideration and analyzed using the same statistical method. Numerical data are expressed as medians at each time point. All statistical analyses were performed with Stata 10.0 software (StataCorp, College Station, TX), and* P*<0.05 was considered statistically significant.

## 3. Results

### 3.1. Patient Demographics and Clinical Data

**Figure 3:**
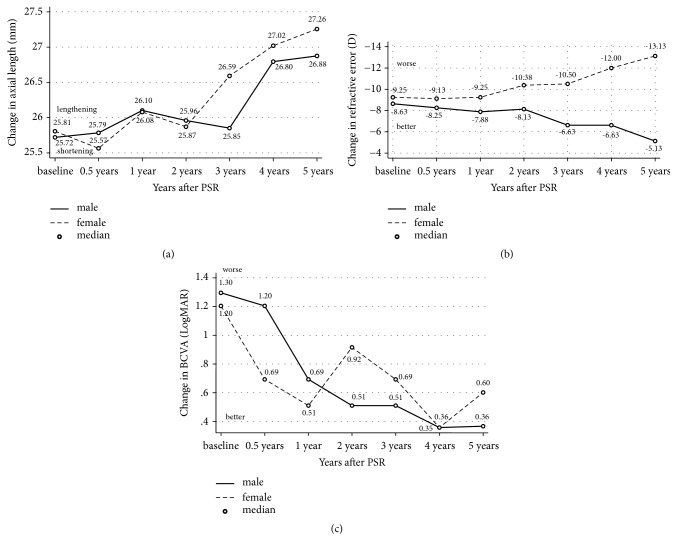
*Axial length, refractive error, and BCVA of the 5-year follow-up.* (a) The representative picture showed the trend of axial length of the 5-year follow-up. It generally displayed an upward trend in both male and female children. (b) The representative picture showed the trend of refractive error of the 5-year follow-up. It generally displayed a downward trend in male children, while it showed an upward trend in female children. (c) The representative picture showed the trend of BCVA of the 5-year follow-up. It generally displayed a downward trend in male children, while it was tortuous in female children.

A total of 47 children (17 females and 30 males) were recruited for our study, and their ages ranged from 3 to 15 years (mean age, 6.3±3.6 years). In total, 50% of the children (20 children, 40 eyes) participated in the 6-month review, 41% of the children (17 children, 33 eyes) participated in the 1-year review, 26% of the children (11 children, 21 eyes) participated in the 2-year review, 16% of the children (7 children, 13 eyes) participated in the 3-year review, 13% of the children (5.3 children, 11 eyes) participated in the 4-year review, and 8% of the children (3.3 children, 7 eyes) participated in the 5-year review. The observed trends in axial length, refractive error, and BCVA are shown in Figure 3. The follow-up period was 5 years.

### 3.2. Axial Length

There was a statistically significant difference in axial length between preoperative and postoperative measurements (*P*<0.05), in detail 6-month (*P*=0.003), 1-year (*P*=0), 2-year (*P*=0), 3-year (*P*=0), 4-year (*P*=0), and 5-year (*P*=0). The average value for axial elongation was 1.32 mm over 5 years, or approximately 0.26 mm per year. According to the mixed model tests, axial length elongation was correlated with baseline age in children who underwent PSR surgery (*P*<0.05). With regard to gender, a statistical analysis found that there was no significant difference (*P*=0.09), indicating that gender was not related to axial length.

### 3.3. Refractive Error

Comparisons between preoperative and postoperative measurements showed that there was no significant difference in refractive error at the follow-up visit performed after the surgery (*P*<0.05), in detail 6-month (*P*=0.51), 1-year (*P*=0.50), 2-year (*P*=0.46), 3-year (*P*=0.56), 4-year (*P*=0.30), and 5-year (*P*=0.16). According to the mixed model tests, changes in refractive error were associated with gender (*P*<0.05) but not baseline age (*P*=0.34). This test revealed that, in boys, refractive error was relatively steady in the following years, whereas, in girls, it increased with age.

### 3.4. Best-Corrected Visual Acuity

At the follow-up performed after the surgery, we found that there were significant differences in BCVA between preoperative and postoperative measurements (*P*<0.05), in detail 6-month (*P*=0), 1-year (*P*=0), 2-year (*P*=0), 3-year (*P*=0), 4-year (*P*=0), and 5-year (*P*=0). BCVA improved in 71 eyes (83.52%) and remained stable in 14 eyes (16.47%), and none of the records indicated a visual decline in any postoperative patient. BCVA was markedly better at the final follow-up than before the operation. Mixed model tests found that there was an association between BCVA and baseline age in children who underwent PSR surgery (*P*<0.05), with younger children achieving a better prognosis. There was no statically significant difference in BCVA and gender according to the same analysis (*P*=0.94).

### 3.5. Anatomical Outcomes

No eye movement restriction or strabismus was found in postoperative extraocular muscle examinations. Conjunctival congestion and edema were observed in all of the children who underwent scleral reinforcement at an early stage, and both conditions were alleviated after several weeks. Postoperative infection was not found in a slit-lamp check in any of the children. No postoperative pathological signs were found in any eye during the fundus examination performed at the last visit. There were no cases of diplopia, hemorrhage, or IOP elevation. None of the eyes lost visual acuity because of the surgery or during the follow-up period.

## 4. Discussion

High myopia is characterized by constant axial elongation and gradual thinning of the posterior sclera, both of which are likely due to the weakening of the biomechanical properties of the sclera [21, 22]. This condition occurs in the early stage of life, carries a high risk of predisposing young children to prevalent amblyopia, and can cause the retina and choroid to degenerate in adulthood [23]. PSR was first proposed by Shevelev in 1954 [24–26]. Later, in 1963, Borely suggested that there is an X, Y band type of PSR [12, 27, 28]. PSR is believed to slow axial elongation by directly affecting the mechanical force of the reinforcement band surrounding the posterior pole and/or by affecting the sclera remodeling and hyperplasia induced secondarily to the nonspecific inflammatory reaction that occurs between the posterior sclera and the reinforcement band [29]. PSR is also thought to promote neovascularization of the sclera, thus inducing the formation of a new vasoganglion and improving the microcirculation within the sclera, choroid, and macula [30, 31]. Because of these advantages, PSR might be the most effective method for delaying or stopping the progression of high myopia.

The most critical component of the PSR procedure is that the reinforcement band must precisely surround the posterior pole and be anchored in place to the sclera. If these steps are not successfully performed, the expected effect could be reduced or unfavorable results achieved. Additionally, the magnitude of the strength produced by the enforcement band depends on the material used. Previous studies have demonstrated that a homologous human scleral strip is the best choice for the PSR procedure [32–36]. The Snyder-Thompson PSR reinforces a single band of donor sclera (60 mm in length and 8 mm in width) when placed underneath the medial, inferior, lateral, and superior rectus and wrapped around the posterior pole of the eyeball. This is the most widely used PSR technique in clinics, and the literature shows that this approach achieves good therapeutic effects; however, its disadvantages are clear. Obtaining sufficient operational skill to successfully perform a Snyder-Thompson PSR requires a long time. Additionally, because the scleral band crosses four major extraocular muscles, the relatively severe surgical trauma inflicted by this procedure may cause a great deal of suffering in children [12, 28]. The Nurmamedov NN PSR strengthens the eyeball by fixing four scleral grafts (8 x15 mm) to the internal areas of the four rectus muscles [37–39]. This type of PSR demands fewer surgical skills and is therefore easier for surgeons to learn. The benefit of these scleral grafts is that they do not touch the extraocular muscles and therefore cause little damage. However, the curative effect of this procedure is limited because the grafts do not directly strengthen the posterior pole of the eyeball [12]. The literature has reported that the Nurmamedov NN PSR has a poor curative effect when used to treat severe high myopic eyes with posterior scleral staphyloma [40]. Compared to previous procedures, our modified PSR is a unique method. First, the surgeon separately places two parts of the half-round scleral buckle across the inferior oblique and lateral rectus. The surgeon therefore causes less surgical damage than is observed in other procedures. Second, because this type of PSR does not disturb the other three rectus muscles and reduces the need for suture fixations, the surgical skills needed to perform it are simpler and require a relatively short learning time to master. Third, each half-round scleral buckle covers the posterior pole, which corresponds to the peripheral macula, thus ensuring the therapeutic effect of the procedure.

In our current study, the results show that the average axial elongation was 0.26 mm per year over the 5-year follow-up period. This change is equivalent to an increase in refractive error of -0.78 D according to calculations performed using a Gullstrand 1 schematic eye. Gerinec et al. reported a mean increase in axial length of 0.2 mm per year after PSR during a follow-up of 3 to 5 years [12]. Minjie et al. obtained a significant result, with the PSR group showing a 0.25 mm increase in axial elongation per year (4.48±1.3-year follow-up) and the control group showing a 0.46 mm increase per year in children aged 6.50±3.23 [4]. Zhao's report performed PSR on high myopic children whose age ranged from 4 to 6. Their study showed that axial length increased by approximately 0.20 mm per year over a 3-year period after PSR, similar to findings in normal school children (0.25 mm per year). The similar result was also reported by B Ward in a study with a 5-year follow-up after PSR with a 0.2 mm yearly increase of axial length [5, 23]. In this study, the modified PSR achieved results similar to those described in previous studies. Our outcome demonstrates that axial elongation and myopic progression are delayed by PSR and that a new surgical method introduced here achieved curative effects similar to those described in previous studies of other approaches. Mixed model tests confirmed that there was a negative relationship between axial length elongation and baseline age. Our results show that BCVA was significantly better postoperatively than preoperatively. In total, 71 eyes (83.52%) improved, 14 eyes (16.47%) remained stable, and none were recorded as showing a visual decline. Previous reports have described similar findings. These include Xiu et al., who performed a study of the modified Snyder-Thompson PSR in which they included 52 eyes with a 5-year follow-up and found that BCVA improved in 12 eyes (23.08%), was unchanged in 37 eyes (71.15%), and was worse in 3 eyes (5.77%) [29]. In their study, the inclusion criteria were a refractive error ≥-12.00 D and an axial length ≥28 mm. Hence, the conditions observed in their patients were worse than those observed in ours. Similar results were also reported by Zhao et al. and Minjie et al., who observed significant improvement in the BCVA of patients who underwent PSR with serious complications [4, 23]. There were two possible reasons for the postoperative improvements in BCVA observed in our study. One is that our inclusion criteria limited axial elongation and myopia progression, and this may have prevented the deterioration of vision; and the other is the natural development of vision in children.

The results showed that there was no significant difference in refractive error between measurements taken before and after this modified PSR was performed. This was due to the contribution of the scleral buckle, which slowed down axial length elongation, preventing it from pathologically increasing. A regression analysis indicated that refractive error was not correlated with the distribution of follow-up years after the surgery but was associated with gender. In Zhang's report, the prevalence of myopia was relatively high in girls, and this difference might be associated with differences in outdoor activities between boys and girls [41]. In our study, we included 30 males and 17 females, and the results of mixed model tests might have been affected by selection bias.

In our study, no severe adverse events were observed after the PSR. Normal postoperative responses, such as conjunctival congestion and edema, were present in previous studies of the Snyder-Thompson PSR [4, 5, 22]. Hence, the modified PSR described here is a safe method for treating highly myopic children.

Despite the favorable outcomes observed for PSR in the present study, several limitations should be mentioned. These limitations included the retrospective design of the study, the lack of a control group, loss to follow-up, and the relatively small number of enrolled children. All statistical analyses were performed for the above-described defects, and our data confirm that this operation is effective. Future studies should include a control group, a larger sample population, and closer follow-up or should be performed with a prospective design to more precisely compare the effects of these surgical methods in the future.

In conclusion, our findings suggest that this modified PSR has the potential to arrest axial elongation in eyes with high myopia. It is a safe operation that requires fewer operational skills and causes little trauma. Further studies are needed to evaluate the long-term safety and effectiveness of this surgery.

## Figures and Tables

**Table 1:** Demographic and clinical characteristics.

PSR7 (n=47)	
Age (years)	6.3±3.6
Range	3-15
Sex:	n (%)
Male	30 (64.0)
Female	17 (36.0)
Follow-up period (years)	1.80±1.44
Range	0.5-5

**Table 2:** Comparisons of axial length, refractive error, and BCVA between before and after PSR for 6 months and 1, 2, 3, 4, and 5 years.

	Axial length (mm)	Refractive error (D)	BCVA (log⁡MAR)	Number of children	Number of eyes	Proportion
Baseline	25.77	-9.00	1.20	47	85	100%
6 months	25.77	-8.50	1.20	20	40	50%
1 year	26.09	-8.50	0.69	17	33	41%
2 years	25.96	-8.25	0.69	11	21	26%
3 years	26.01	-7.50	0.69	7	13	16%
4 years	26.94	-7.88	0.36	5.3	11	13%
5 years	27.08	-6.38	0.51	3.3	7	8%

## Data Availability

The data used to support the conclusions of this study are available from the corresponding author upon request.
